# Discovering research articles containing evolutionary timetrees by machine learning

**DOI:** 10.1093/bioinformatics/btad035

**Published:** 2023-01-17

**Authors:** Marija Stanojevic, Jovan Andjelkovic, Adrienne Kasprowicz, Louise A Huuki, Jennifer Chao, S Blair Hedges, Sudhir Kumar, Zoran Obradovic

**Affiliations:** Center for Data Analytics and Biomedical Informatics, Computer and Information Science Department, Temple University, Philadelphia, PA 19121, USA; Center for Data Analytics and Biomedical Informatics, Computer and Information Science Department, Temple University, Philadelphia, PA 19121, USA; Department of Biology, Temple University, Philadelphia, PA 19121, USA; Department of Biology, Temple University, Philadelphia, PA 19121, USA; Center for Data Analytics and Biomedical Informatics, Computer and Information Science Department, Temple University, Philadelphia, PA 19121, USA; Department of Biology, Temple University, Philadelphia, PA 19121, USA; Institute for Genomics and Evolutionary Medicine, Temple University, Philadelphia, PA 19121, USA; Department of Biology, Temple University, Philadelphia, PA 19121, USA; Institute for Genomics and Evolutionary Medicine, Temple University, Philadelphia, PA 19121, USA; Center for Data Analytics and Biomedical Informatics, Computer and Information Science Department, Temple University, Philadelphia, PA 19121, USA

## Abstract

**Motivation:**

Timetrees depict evolutionary relationships between species and the geological times of their divergence. Hundreds of research articles containing timetrees are published in scientific journals every year. The TimeTree (TT) project has been manually locating, curating and synthesizing timetrees from these articles for almost two decades into a TimeTree of Life, delivered through a unique, user-friendly web interface (timetree.org). The manual process of finding articles containing timetrees is becoming increasingly expensive and time-consuming. So, we have explored the effectiveness of text-mining approaches and developed optimizations to find research articles containing timetrees automatically.

**Results:**

We have developed an optimized machine learning system to determine if a research article contains an evolutionary timetree appropriate for inclusion in the TT resource. We found that BERT classification fine-tuned on whole-text articles achieved an F1 score of 0.67, which we increased to 0.88 by text-mining article excerpts surrounding the mentioning of figures. The new method is implemented in the TimeTreeFinder (TTF) tool, which automatically processes millions of articles to discover timetree-containing articles. We estimate that the TTF tool would produce twice as many timetree-containing articles as those discovered manually, whose inclusion in the TT database would potentially double the knowledge accessible to a wider community. Manual inspection showed that the precision on out-of-distribution recently published articles is 87%. This automation will speed up the collection and curation of timetrees with much lower human and time costs.

**Availability and implementation:**

https://github.com/marija-stanojevic/time-tree-classification.

**Supplementary information:**

Supplementary data are available at *Bioinformatics* online.

## 1 Introduction

Text mining has the potential to reshape the discovery of research articles containing domain-specific knowledge from the fast-expanding corpus of scientific literature. One such area is evolutionary biology, in which the growing affordability of genome sequencing technology has revolutionized the assembly of the tree of life. Hundreds of new peer-reviewed articles containing timetrees, phylogenetic trees scaled to time, are published yearly in many journals (Kumar *et al.*, 2022). Timetrees are present in published articles in graphical formats (images) that display hierarchical trees of species. These results are not always accessible by text searching when looking for divergence times of species of interest and the articles containing relevant divergence times. The TimeTree of Life (TToL) project, initiated in 2004, has been locating research articles containing timetrees, acquiring timetrees, standardizing timetrees and conducting their meta-analysis to produce a global timetree (Hedges *et al.*, 2006; Kumar *et al.*, 2022). In the most recent release, the global timetree contains >135 000 species assembled from constituent timetrees from >4000 published articles (Kumar *et al.*, 2022). This information is delivered through a user-friendly web resource cited thousands of times. Many students and the general public also use the TToL website, evidenced by >250 000 database queries annually.

Since 2004, curators of the TimeTree (TT) database have been manually identifying relevant research articles. A combination of keywords is used to search the Google Scholar (GS) and PubMed archives, which produce a list of articles via the web. Then, curators scan article titles to identify potential timetree articles, which is time-consuming, expensive and eye-straining. This scanning is followed by clicking the PDF/text-download link of promising articles, another time-consuming step, followed by a manual inspection of the article's content. When an article contains a timetree, it is retained for further processing. For almost two decades, the curators have downloaded and inspected titles of thousands of articles and manually scanned 14 366 downloaded full-text articles to look for timetrees. Of these, 4292 research articles were found to contain timetrees, which is a manual true positive rate of 29.88%. Those timetrees became candidates for curation into the TT database (www.timetree.org). This approach of locating relevant research articles is tedious, time-consuming and error prone because a timetree in an article is not entirely predictable based on its title or abstract alone.

**Fig. 1.**
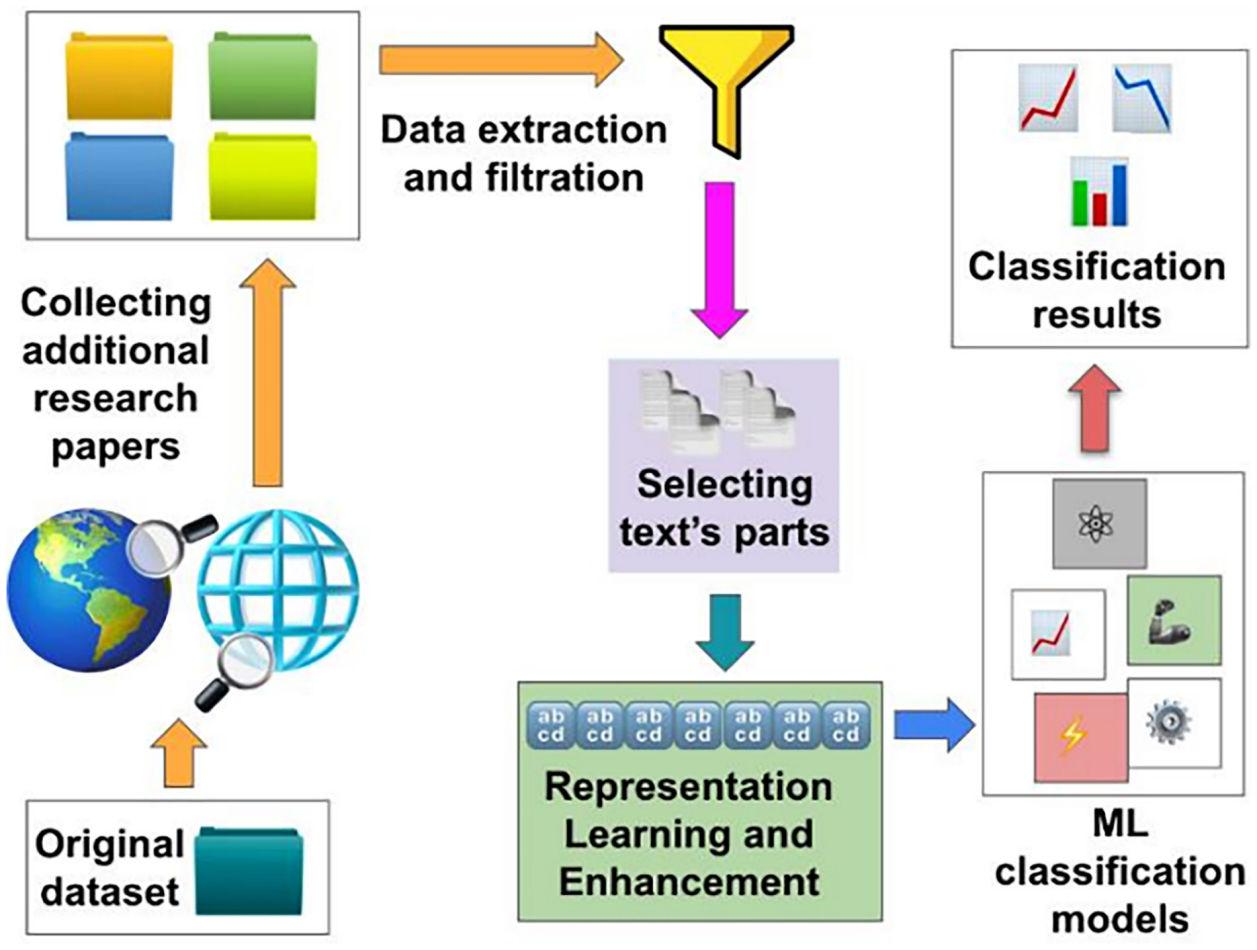
Overview of the proposed TimeTreeFinder (TTF) system, including searching, collecting and analyzing research articles to discover timetree-containing articles

The TT curators did not use text-mining tools, except for internet searching, to find research articles containing timetrees because there were no tools for processing the corpus of scientific articles to discover evolutionary knowledge. While many computational text-mining approaches and tools have been applied extensively in medical and molecular biology research, our survey revealed no investigations of their usefulness for retrieving articles containing evolutionary trees and guidelines on the best tools and their domain-specific optimization. Therefore, the main goal of this study was to investigate the performance of available simple and advanced text-mining techniques to identify timetree-containing articles. The new TimeTreeFinder system (TTF; Fig. 1) processes the corpus of relevant scientific literature accessible from resources such as GS, PMC Open Access (PMC-OA) Database, PMC Historical, bioRxiv and scientific journals' websites.

In the following, we present the results of the implementation and analysis of TTF that uses TFIDF (Jones, 1972; Luhn, 1957), Lasso (Tibshirani, 1996) and word2vec (Mikolov *et al.*, 2013a, b) algorithms for constructing Google search queries and state-of-the-art machine learning algorithms such as BERT (Devlin *et al.*, 2018), BioBERT and SciBERT for detecting the presence of timetrees in research articles. BERT-based state-of-the-art models can process up to 512 words, so we trained them to classify research articles using only titles and abstracts in the same way as used by manual curators of the TT project.

As shown below, recognizing timetree-relevant papers solely from title and abstract was the least effective approach (see results). This problem prompted us to test the hypothesis that more accurate retrieval is possible by analyzing full-text articles. While it improved results, it still produced many errors. The full text can be misleading to classification methods because many articles contain informative phrases, but no timetrees are present in the article. We hypothesized that models would be more accurate if trained and applied to carefully excerpted texts around the figures because timetrees are usually present in the image form in the research articles.

In the following, we discuss the created TTF tool that ultimately achieves an F1 score of 0.88 in classification despite the high dimensionality of the problem. We also investigated machine learning models and features (word and word pairs) essential for finding articles containing timetrees accurately. Finally, we discuss how the system presented can be adapted to collect relevant data and discover meaningful information from various texts (e.g. grants proposals, reports, research papers, web pages and news) for other scientific and non-scientific applications.

## 2 Materials and methods

The new automated system, TimeTreeFinder (TTF), to search, extract and analyze the corpus of scientific literature and individual articles to identify relevant articles is outlined in Figure 1.

### 2.1 The ground truth TT dataset

Over the last 20 years, the TT project staff has manually scanned 14 366 articles and labeled them. The articles were labeled as positive (4292) if they contained tables, figures or descriptions of molecular divergence times. Otherwise, they were given a negative label (10 074). This dataset was used in all our analyses and experiments as ground truth.

### 2.2 Processing the corpus of scientific literature

We retrieved scientific articles from four online resources for this project: GS; journals that published most of the articles in the TT dataset; PMC-OA subset; PMC Historical dataset (https://www.ncbi.nlm.nih.gov/pmc/tools/textmining/); and bioRxiv machine access and text/data mining resources (https://www.biorxiv.org/tdm). These online resources are massive, and it is time- and resource-consuming to download and analyze PDF files and other information about millions of articles, even when done automatically. Therefore, we searched for and downloaded only pertinent articles when possible (GS). In addition, we filtered out irrelevant articles from all sources.

The approach taken to process the GS dataset was as follows. We first determined representative phrases (words and word pairs) from the ground truth TT dataset. We used TFIDF (Jones, 1972; Luhn, 1957) to create features (words and word pairs) and L1-regularized logistic regression, LASSO (Tibshirani, 1996), to classify articles from the TT dataset. Lasso suppresses irrelevant features and gives positive coefficients to the most important phrases for detecting research articles containing timetrees. This list of phrases, called PL, is provided in the Supplementary Material. Then, we applied the word2vec algorithm (Mikolov *et al.*, 2013a, b) to identify additional informative words used frequently in the same contexts as the PL words. The resulting collection of phrases was filtered by frequency to remove rarely occurring combinations and misspellings. The resulting list of features, WL, was added to PL to form the final list of phrases, FL.

We designed two types of queries using phrases from FL. The FL-simple query utilized one phrase, and the FL-complex query used a logical combination of phrases. These efforts retrieved 5070 articles from the FL-simple query and 8013 articles from the FL-complex query. While we expected a much larger number of articles, GS's restrictive policies forbid downloading too many PDFs, and our university's library could not access all the journals. However, we can run the automatic search again and collect the articles when they become available. Details on GS search are provided in the Supplementary Material.

Unlike GS, which provides a search engine, we needed to develop *ad hoc* approaches for collecting articles from all the other resources (see Supplementary Material). In this case, we downloaded all available PDFs without imposing any filtering by keywords. Naturally, we downloaded a large number of articles, which ranged from 117 000 articles from biological journals to 4.17 million articles from PMC-OA. Next, we filtered them to reduce irrelevant articles using keywords (see Supplementary Material). This procedure removed 51% of the articles downloaded from journals, 90% of PMC-OA articles, 94% of PMC Historical articles and 75% of bioRxiv articles. The resulting collections were candidates for finding timetrees. These data collections were then subjected to representation learning and classification analysis.

### 2.3 Representation learning

We compared six representation learning methods: TFIDF (Jones, 1972; Luhn, 1957), doc2vec (Le and Mikolov, 2014), BERT (Devlin *et al.*, 2018), SciBERT (Beltagy *et al.*, 2019), DistilBERT (Sanh *et al.*, 2019) and BioBERT (Lee *et al.*, 2020). Doc2vec is a method that uses a small neural network to learn the vector representation of sentences and paragraphs. TFIDF creates a matrix in which rows represent document vectors, columns represent word vectors and values are TFIDF scores for each word-document combination. An advantage of TFIDF is that it aims to capture information from the entire document instead of relying on a local context learned by doc2vec. Research articles are long texts with possible distant dependencies; therefore, we hypothesize that doc2vec is not powerful enough to represent such long texts. BERT-based models learn from the whole sequence, like TFIDF, but they can learn from sequences of up to 512 words. We considered SciBERT and BioBERT as they specialize in the representation of research articles and biological articles, respectively. Finally, we tested DistilBERT as a lighter and faster version of BERT known to achieve similar performance (Sanh *et al.*, 2019).

### 2.4 Representation enhancing

Since representation is learned separately from classification, labels are not required for that step. We integrated the ground truth TT dataset and downloaded filtered datasets for representation learning to benefit from more data and contexts. We ran classification models on representations learned just from the TT dataset and representations learned from all data to test if using additional data to learn representation improves classifications.

### 2.5 Classification

Research articles are complex and high-dimensional data, and it is hard to form hypotheses about their classification. Therefore, we compared nine diverse but commonly used classification methods: L1-norm-regularized logistic regression (L1), decision tree (DT), random forest (RF), k-nearest neighbors (KNN), support vector machines (SVM), adaptive boosting (Ada), gradient boosting (GB), bagging (Bag) and classification layers of BERT-based methods (BERT, DistilBERT, SciBERT, BioBERT). They represent different types of classification algorithms with various desirable properties, enabling us to explore which approaches perform best at classifying the biological domain of timetrees.

### 2.6 Evaluation

We investigated the classification results of models to understand when errors were made. The models that performed poorly missed a lot of relevant articles (type II error) and kept a lot of irrelevant articles (type I error). As curators need to review the data from all research articles predicted as positive, the objective is to reduce type I error to decrease human effort. We also wanted the smallest possible type II error as the main goal is to identify as many articles containing a timetree as possible. We used the F1 score (range 0–1) as the main metric since it is maximized when both types I and II errors are minimized. We fine-tuned each model using a grid search of hyperparameters and selected those with the best F1 score on the cross-validation dataset. Details are described in the Experimental Settings paragraph in the Supplementary Material. Documents predicted as positive were checked by human curators to ensure that the predictions contain a timetree and that the analysis of empirical datasets produces it.

### 2.7 Generation of text excerpts datasets

We found machine learning models to make three main types of errors in misclassified articles. The first mistake was identifying articles that only described methodology, especially software used for visualization, but did not contain any data on new timetrees. Second, many false positives discussed geological periods or species but lacked information on molecular divergence times. Third, research articles describing the time of divergence of species in small parts of the text were wrongly excluded (false negatives).

We hypothesized that these issues would be reduced by selecting relevant information from articles and classifying articles based on that information instead of the whole text. This technique has two benefits: (i) shorter texts are easier for machine learning models to comprehend and (ii) we automatically exclude irrelevant text in search of a timetree (e.g. introductions, references and other descriptions).

Since timetrees are usually displayed in figures, we selected figure captions as well as parts of research articles that mention figures. We considered both figures described and shown in the main text and the supplement of the research articles. We used newline spacing to understand when the paragraph or caption mentioning figures starts or ends. Additional details are provided in the Supplementary Material.

To understand how much text context is needed for a model to learn if the figure contains a timetree, we compared the performance using 800 characters and only 300 characters around the place where a figure was mentioned. Then, we followed the process described in representation learning, representation enhancing, classification methods and evaluation using datasets containing text excerpts and short text excerpts instead of whole-text datasets.

### 2.8 TTF tool

All the methods mentioned above are implemented in the TTF tool. It supports searching, collecting, extracting, filtering and normalizing data from relevant sources of phylogenetics research articles. The TTF tool is also used for training representation learning and classification methods on research articles from the TT dataset. In addition, it implements the selection of short and long text excerpts relevant to timetrees and representation and classifier learning over those data. Finally, the TTF tool validates learned models using the TT dataset, which was set aside from training data and evaluated performance on newly collected datasets. A detailed description of the training and validation split and other experimental settings can be found in the Supplementary Material.

## 3 Results and discussion

This section presents results obtained using TTF, which automates search, collection, filtering and timetree discovery in the research articles described in Section 2. We discuss the properties of collected datasets and how they compare to the original data. Then, we compare scores from different machine learning techniques for data representation and classification and propose techniques for selecting relevant text excerpts from papers.

### 3.1 Most common words in the TT dataset

**Fig. 2.**
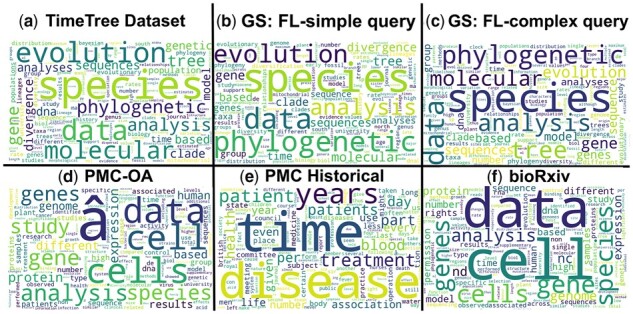
Frequencies of most common words in (**a**) TimeTree dataset, Google Scholar datasets from (**b**) FL-simple query and (**c**) FL-complex query, (**d**) PMC-OA, (**e**) PMC Historical and (**f**) bioRxiv dataset

**Fig. 3.**
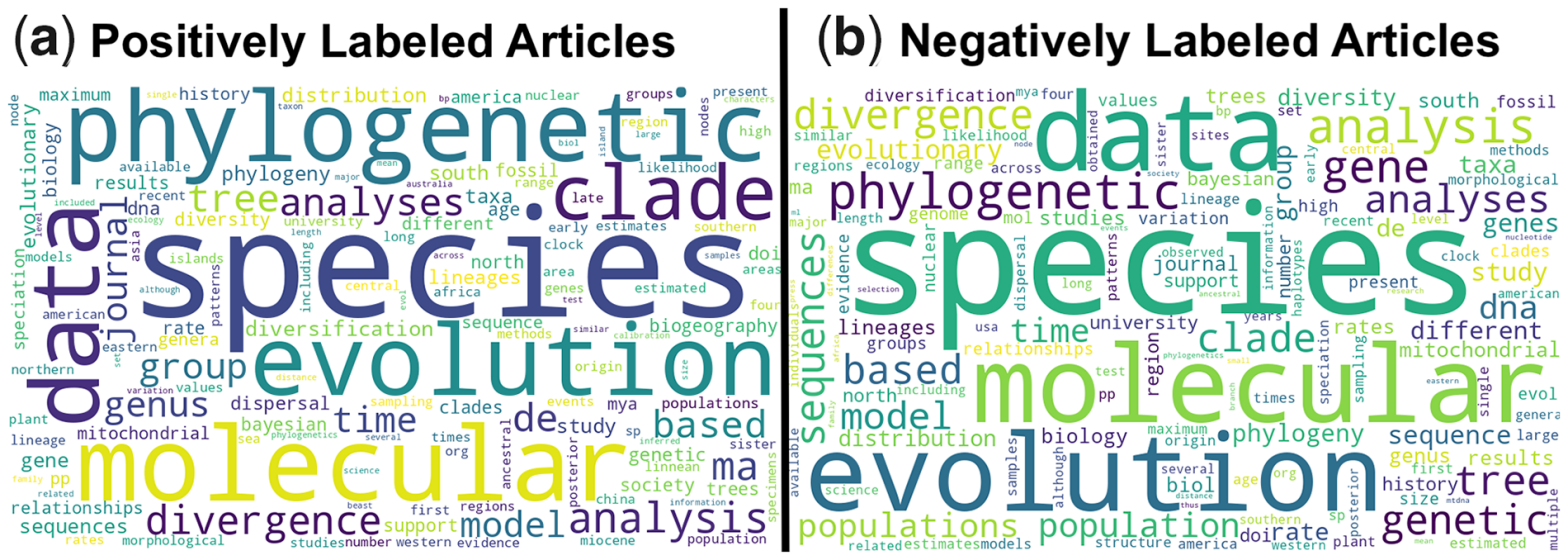
Comparison of the distributions of the most common words among papers labeled as (**a**) positive and (**b**) negative in the TimeTree dataset. There is quite a bit of overlap between the two sets

Figure 2a shows the cloud of the most common words in the ground truth manually curated TT dataset. The size of the words corresponds to their frequency in the TT dataset. The most common words are *species*, *evolution*, *molecular*, *phylogenetic*, *data*, *gene*, *analysis*, *tree*, *genetic*, *sequence*, *clade* and *divergence*. We excluded generic stop words and other common words irrelevant to phylogenetics (see Supplementary Material) from the word cloud figures (Figs. 2 and 3).

### 3.2 Properties of collected and filtered datasets

This section investigates the properties of datasets collected through the process described in Section 2. Figure 2b and c represents the distributions of the most prominent words in datasets obtained from GS by FL-simple and FL-complex queries. Those datasets show extensive overlaps with the TT dataset. This similarity means that classifiers learned using the TT dataset would perform well on GS datasets, saving effort in finding timetree-containing articles. Datasets collected from journals, PMC-OA and bioRxiv are available only in bulk and could not be searched for relevant articles. Therefore, we created a filtering step to select relevant papers. Filtering removes articles that do not contain at least one of the most relevant timetree words (see the full list in the Supplementary Material). This filtering system was tested on the TT dataset to ensure it performs well. None of the positively labeled documents in the original dataset were removed, i.e. the false negative rate was zero. Only 9.6% of TTD papers in the true negative set were not retained. Therefore, the filtering system works well.

We applied the filtering system to all the datasets. Most articles were filtered out from the PMC Historical datasets (only 6% retained) because they contain articles on many topics unrelated to phylogenetics and timetrees. This dataset also contained phylogenetics articles published before the 1990s, which did not have information on timetrees because the field has grown much in the last three decades. The PMC-OA dataset, while still heavily focused on medicine, retained 10% of the article collection since it is more recent and contains more phylogenetic information. The filtering retained 25% of the bioRxiv articles as bioRxiv focuses on biology and contains many more recent research articles (since 2013). In the last two decades, the publication of timetrees has been more common and driven by the analysis of molecular datasets. In processing research articles from selected journals, 50% were retained because we collected only research articles from relevant journals.

Common words in the PMC-OA, PMC Historical and bioRxiv datasets are shown in Figure 2c–f, respectively. Their comparison with the common words of the TT dataset reveals that PMC and bioRxiv datasets are not very similar to the ground truth despite filtering. Some TT dataset frequent words, such as *data*, *species* and *analysis*, are common in bioRxiv as well, but many medical words which are not common in the TT dataset frequently occur in PMC datasets.

### 3.3 Classification models and their predictions

**Fig. 4.**
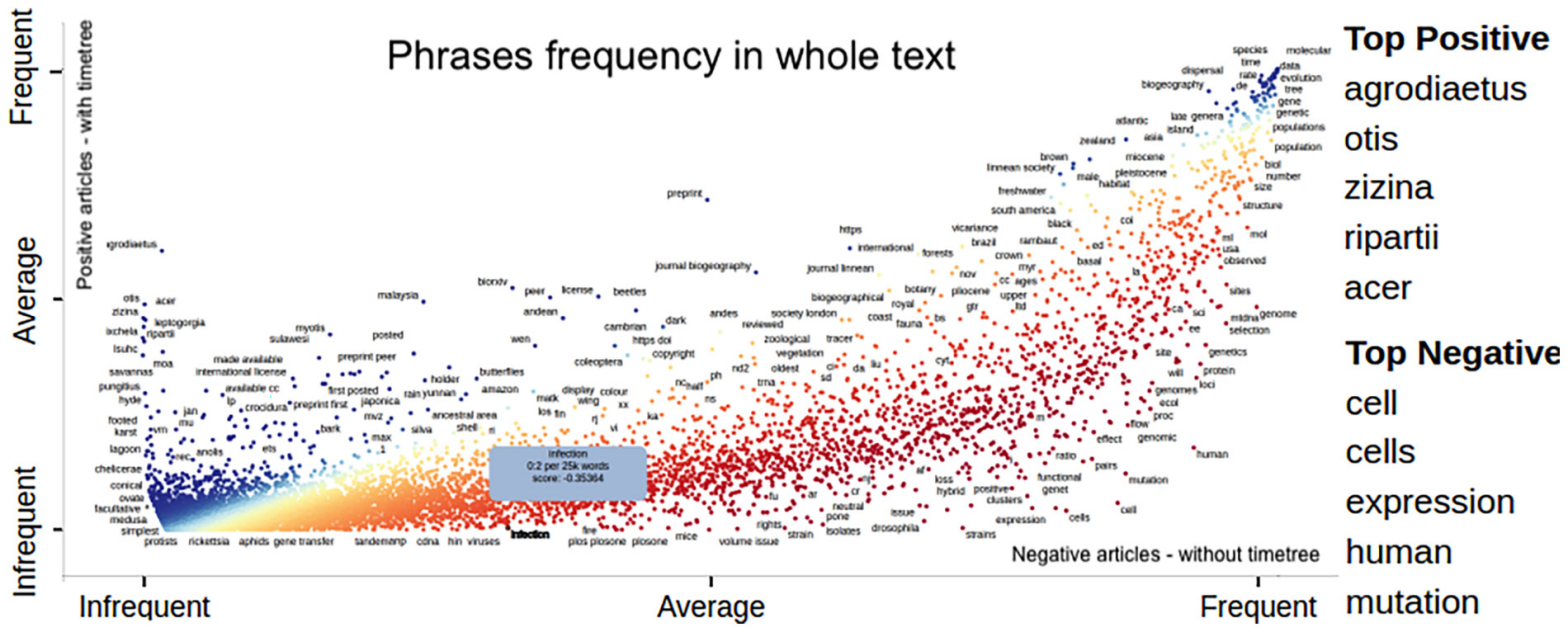
Scatter plot of words from articles in original datasets. The horizontal axis represents word frequency in negatively labeled documents and the vertical in positively labeled. The top positive and negative words are displayed on the right. See full-size figure provided in Supplementary Material

Figure 3 shows that papers with positive and negative labels in TT dataset have an almost identical distribution of most common words, pointing to the complexity of the classification task. Figure 4 shows phrases with a positive coefficient in the Lasso model as blue and those with a negative factor as dark red. The *x*-axis displays the frequencies of words in negatively labeled research papers, and the *y*-axis displays word frequencies in positively labeled research papers. Many words are on the diagonal, i.e. they have a similar frequency in positively and negatively labeled papers. The column on the right of the figure shows the words with the most significant positive and negative influence. We conclude that word distribution in positively and negatively labeled research articles are very similar, and classifying those papers is difficult. In fact, classification results show that the best model, achieved with a gradient-boosting classifier over TFIDF features, could produce an F1 score of 0.72 (the second row of Table 1).

**Table 1.** Results from classification analysis

Method	Features from labeled data only		
	Whole text	800-character excerpts	300-character excerpts
L1 + TFIDF	0.711 ± 0.004	0.792 ± 0.012	0.689 ± 0.009
L1 + doc2vec	0.642 ± 0.015	0.750 ± 0.013	0.751 ± 0.018
DT + TFIDF	0.634 ± 0.001	0.757 ± 0.005	0.643 ± 0.010
DT + doc2vec	0.532 ± 0.011	0.616 ± 0.004	0.654 ± 0.039
RF + TFIDF	0.695 ± 0.001	0.839 ± 0.003	0.717 ± 0.009
RF + doc2vec	0.617 ± 0.001	0.750 ± 0.003	0.750 ± 0.007
KNN + TFIDF	0.557 ± 0.019	0.760 ± 0.005	0.608 ± 0.008
KNN+doc2vec	0.582 ± 0.007	0.674 ± 0.037	0.639 ± 0.029
SVM + TFIDF	0.711 ± 0.017	0.817 ± 0.005	0.689 ± 0.001
SVM+doc2vec	0.659 ± 0.019	0.788 ± 0.004	0.754 ± 0.005
Ada + TFIDF	0.673 ± 0.027	0.783 ± 0.010	0.683 ± 0.023
Ada+doc2vec	0.589 ± 0.013	0.720 ± 0.011	0.687 ± 0.015
GB + TFIDF	**0.720 ± 0.007**	0.810 ± 0.002	0.693 ± 0.005
GB + doc2vec	0.599 ± 0.030	0.757 ± 0.023	0.729 ± 0.007
Bag + TFIDF	0.707 ± 0.010	0.812 ± 0.003	0.707 ± 0.005
Bag + doc2vec	0.547 ± 0.013	0.733 ± 0.008	0.728 ± 0.004
Distilled BERT	0.661 ± 0.009	0.873 ± 0.006	0.852 ± 0.002
BERT	0.672 ± 0.001	**0.880 ± 0.003**	0.830 ± 0.044
SciBERT	0.618 ± 0.078	0.837 ± 0.018	**0.864 ± 0.002**
BioBERT	0.657 ± 0.003	0.868 ± 0.001	0.852 ± 0.001

*Note*: Average F1 scores and their standard deviations are shown.

We utilized the best classifier to classify papers in collected datasets so that positive papers could be considered for expanding the TT database. When applying the classifier to datasets collected by searching GS using FL-simple and FL-complex queries, we predicted that 13% and 17.8% of the collection contained a timetree, respectively. Also, 7.8% of the corpus downloaded from relevant journals is predicted to contain the timetrees. Only 0.79% of PMC, 0.00039% of PMC Historical and 1.4% of bioRxiv articles received a positive label.

### 3.4 Improving predictions using text excerpts

**Fig. 5.**
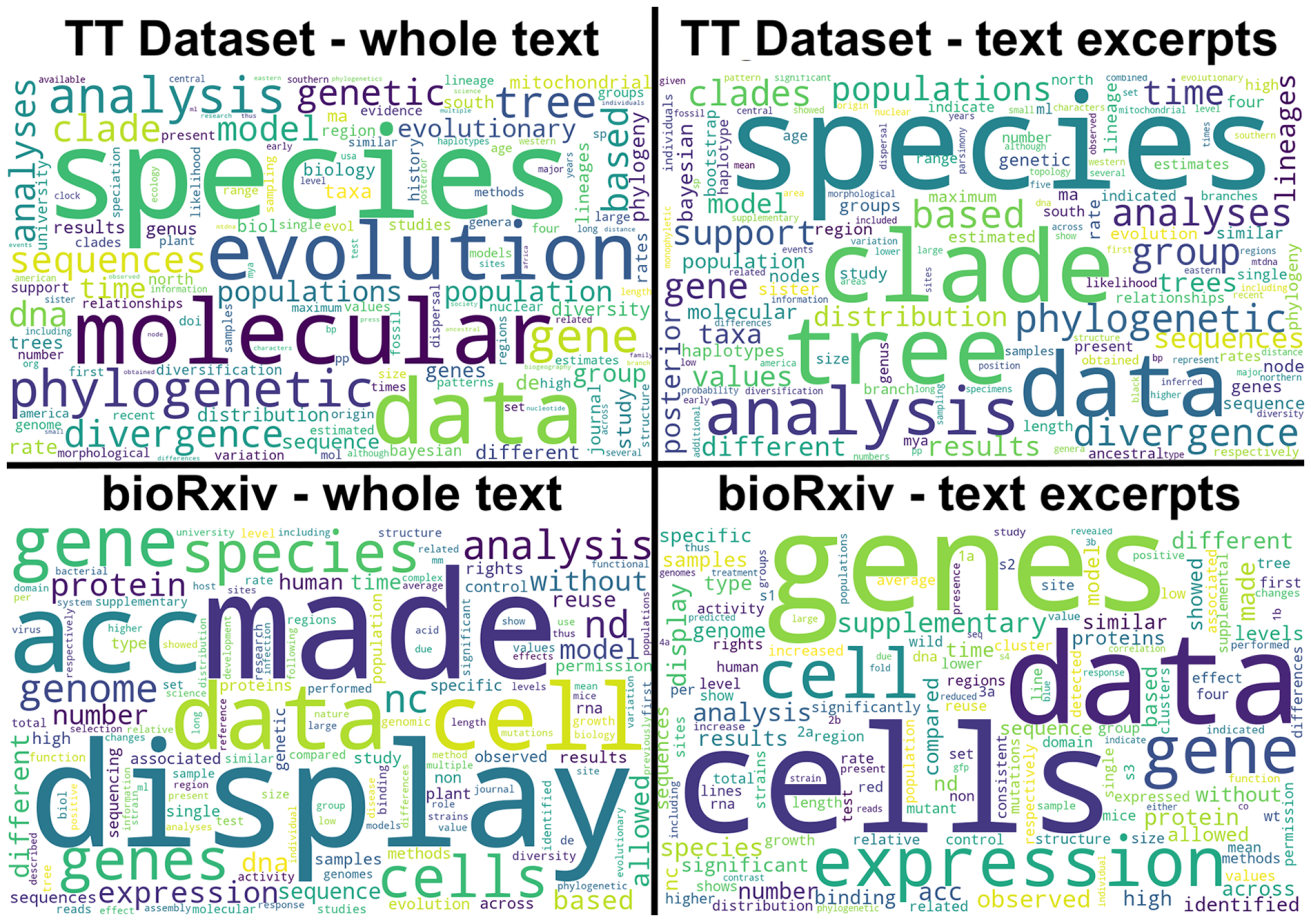
Comparison of distributions of most common words between whole texts (left) and selected texts (right) from two research papers datasets: original (top) and bioRxiv (bottom)

**Fig. 6.**
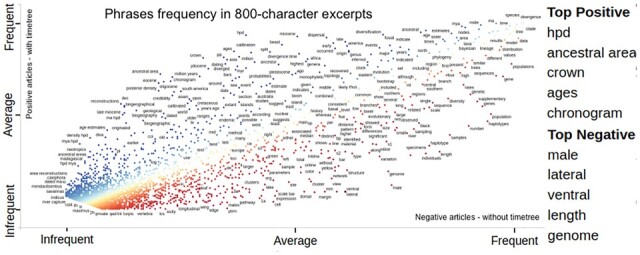
Scatter plot of words in selected texts from articles in original datasets. The horizontal axis represents word frequency in negative documents and the vertical in positive. The top positive and negative words are displayed on the right. See full-sized figure is provided in Supplementary Material

Figure 5 (top) shows that selecting only relevant text excerpts to represent articles from the TT dataset changes the word distribution while keeping the genuinely relevant words *species*, *analysis*, *data*, *divergence* and *tree* as the most common. Keywords for finding timetrees such as *time*, *trees*, *clade*, *analysis*, *analyses* and *distribution* are more prominent in a graph of selected texts excerpts than when using the whole texts. The impact of text excerpts was more significant in the bioRxiv dataset (Fig. 5, bottom). Many unimportant words (e.g. made, acc, display) were eliminated, and some relevant words (such as genes and analysis) became more prominent. Figure 6 has similar content to Figure 4, but it is created for the text excerpts in the TT dataset. It has a different distribution. The points are more scattered, with many more words showing the positive or negative influence and making the problem easier to solve. Also, the list of top positive words in Figure 6 is more relevant to timetrees.

**Fig. 7.**
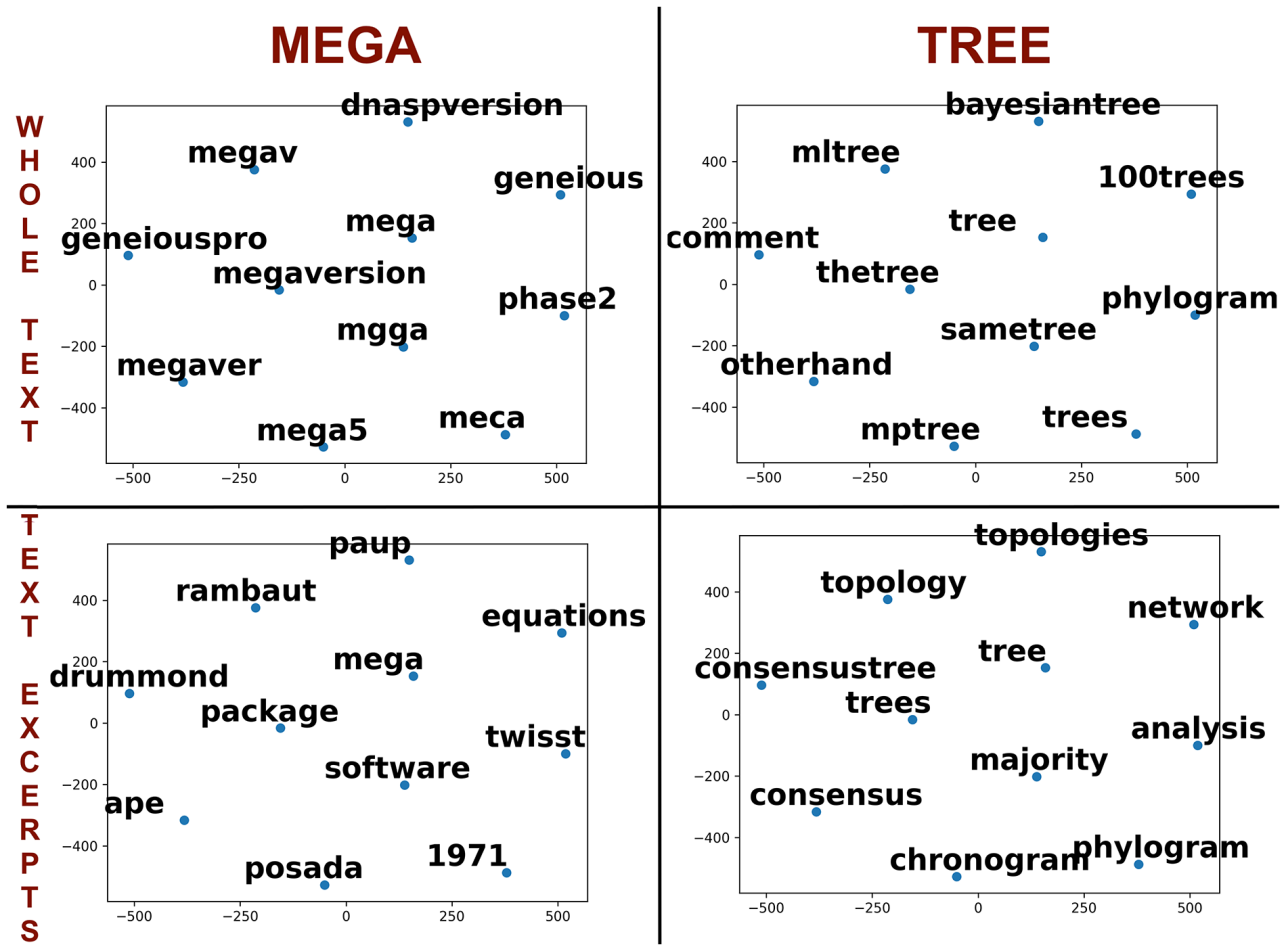
Comparison of projected 100-dimensional embeddings of words in contexts of words *mega* (left) and *tree* (right) learned on the whole papers (top) and on selected texts (bottom)

Figure 7 compares contexts for words *mega* (left) and *tree* (right) learned using the doc2vec algorithm. We display the context of those words as they are truly relevant to molecular timetrees text, but they also have different properties. *MEGA* is a unigram and is the name of a commonly used software, and it is not expected to be used in other contexts in the corpus at hand. “*tree*” is a common word in the English language that can be used in other circumstances, and it is also often used as part of a bigram in phylogenetics (e.g. timetree, molecular tree).

We display the ten most similar words according to the learned vector space, and all four figures show that learned vectors are meaningful. However, there is an apparent difference between the top and bottom graphs. The top graphs mainly contain misspellings, while the bottom graphs contain contextually similar words. This happens because representation learned from selected text excerpts uses only relevant portions of text in which misspellings or errors due to OCR or parsing are fewer. In addition, it is easier to learn the context from shorter selected texts. Consequently, most algorithms perform better on the excerpt dataset (Table 1). For the dataset of 800-character excerpts, BERT produced the best results, followed by the performance for excerpts that were 300 characters long.

Applying the best classifier on text experts from collected datasets, we found that 27.4% and 20.8% of the text excerpts were labeled as positive in the GS FL-simple and FL-complex query datasets, respectively. The journal collection has 11.3% of positive text excerpts. Only 2% of PMC, 1.3% of PMC Historical and 2% of bioRxiv selected text excerpts are predicted as positive. Percentages of positive predictions are larger for classifying the selected text excerpts because no text excerpts contained informative words from many irrelevant papers. Those findings prove that datasets from bioRxiv and PMC sources need to be further filtered using a more complex technique.

### 3.5 Classification results and discussion

Results for different combinations of techniques are presented in Table 1. Columns display the F1 score results of those techniques when representation is learned only on the original dataset. Results with enhanced representation are provided in Supplementary Material. We fine-tuned hyperparameters to optimize for the F1 score as we need to minimize both type I and type II errors. The best classification method for each column is displayed in bold letters. BERT classification using full text achieved an F1 score of 0.672, much smaller than BERT trained on selected text excerpts of length 800, which increases the F1 score to 0.880 with a small standard deviation (0.003).

The best representation and classification methods depend on which parts of the text are used for representation. When the whole text is used, TFIDF representation has the best performance in general, as BERT-based models can look only into the first 512 words, and doc2vec does not have enough capacity to learn from such a long text. When selected text excerpts are used, BERT-like models perform the best. BERT is the best in classifying selected texts of length 800 characters, and SciBERT is the best on selected text excerpts of length 300. DistilBERT, as expected from the literature review, has a 1% worse performance than BERT, but it is twice smaller and twice faster in inference, according to our results. BioBERT was expected to perform better than BERT because of its fine-tuning for biological and medical data, but it does not for any of our datasets. SciBERT was also expected to perform better than BERT in general, as it is fine-tuned on 80% of biological scientific articles. But, its performance is ∼5% worse than BERT's (Table 1). However, it does show excellent performance on short text excerpts.

Enhanced representation does not show a clear improvement in our experiments, although it is usually helpful for doc2vec algorithm learning from the whole text. However, learning from the whole text hurts performance compared to representations learned from text excerpts. The results provided in the Supplementary Material show the discouraging performance of enhanced representation learning, which is probably caused by the considerable difference between distributions of words in the ground truth TT dataset and the target datasets, but further experiments are needed to understand the problem better. TFIDF representation achieves better results than doc2vec representation except when combining doc2vec with enhanced representation on selected texts. It also achieves the best result, F1 = 0.720, when trained on whole texts with gradient-boosting classification. This result is consistent with our hypothesis that TFIDF will perform better due to its ability to learn from the whole document and not the small contexts that doc2vec uses. However, we can also see that when data are shorter (selected texts) and more data are used (enhanced representation), we can achieve close to the best doc2vec results.

### 3.6 The proportion of additional timetree articles predicted

**Fig. 8.**
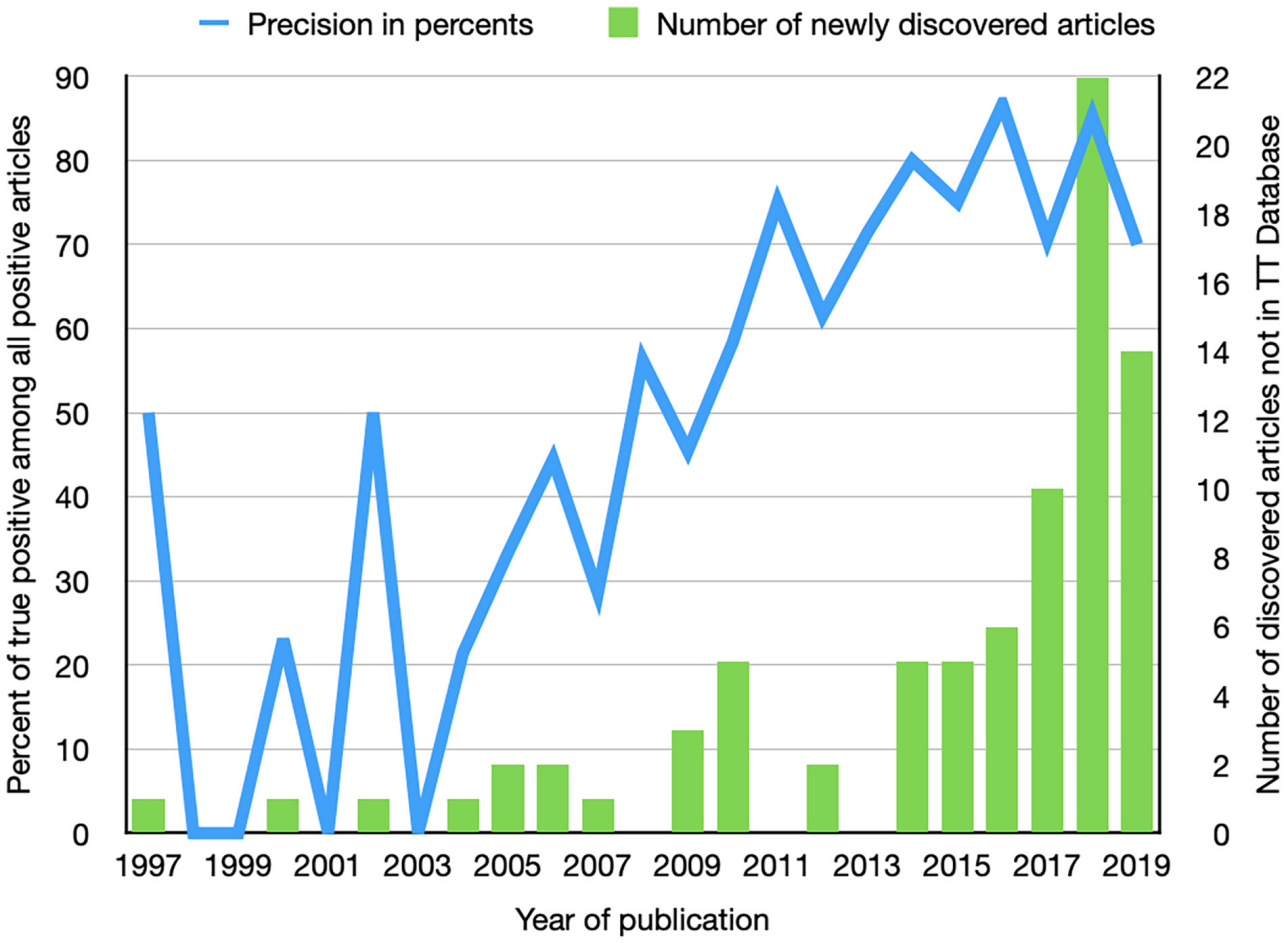
Precision per year of BERT trained on 800-character excerpts and evaluated on. *y* = a proportion of timetree-containing articles in the journals *Molecular Phylogenetics and Evolution*, *Systematic Biology* and *Zoologica Scripta* identified by BERT-800 are missing from the TimeTree database

To establish model precision on out-of-distribution data, we manually examined journal articles predicted as timetree-containing by the best-performing model (BERT with 800-character excerpts). We restricted our assessment to all the articles from three relevant journals (*Molecular Phylogenetics and Evolution*, *Systematic Biology* and *Zoologica Scripta*) because they needed to be manually inspected by the TT curators. BERT, with 800-character excerpts, predicted 304 articles to have a timetree from these three journals. Of these, 163 contained the timetree, i.e. a true positive rate of 53.6%. The blue line in Figure 8 (left *y*-axis) shows prediction precision (true positive among all positive) per year. The TTF accuracy was 87% for recently published articles. Most older articles mislabeled as positives contained a tree but lacked a time component. Therefore, future enhancement of this approach could focus on reducing such false positives for early articles.

Of these true positives, 84 were missing from the *TimeTree* database (51.5%). The fraction of timetrees missing from the TT database shows a temporal pattern, i.e. a greater percentage of articles are missing from recent years (Fig. 8, green bars, right *y*-axis). This is because the number of published articles is increasing, and the cost of manual standardization and assimilation of articles into the TT database is significant. Therefore, the TTF system would help double the number of research articles in the TT database.

## 4 Conclusions

In this work, we created an automatic process for searching, collecting and filtering articles containing molecular timetrees. In addition, we have shown that mining articles represented by text excerpts surrounding mentions of figures achieve better results than all the other alternatives tested. We considered six representation learning techniques and nine classification learning techniques to achieve an F1 score of 0.88 (20.8% absolute improvement over BERT baseline) in selecting articles containing timetrees or divergence times. This system will save considerable time previously devoted to manually scanning full texts and searching the web using keywords. Furthermore, our analyses show that many articles in the published literature are missing from the TT database. Their processing and inclusion will increase the number of species covered and the number of studies per species in the TT database, making it more comprehensive (Kumar *et al.*, 2022).

In addition, the TTF tool can be adapted to search for other phylogenetic information in the scientific literature, such as locating articles containing phylogenies without times and extracting lists of species and other metadata in the selected article. Of course, the TTF models can also be used to parse other relevant literature such as reports, news and blogs. More generally, the TTF tool can be used for searching other documents by training the model using a small labeled dataset with positive and negative labels (ground truth). TTF can extract the most important phrases for finding relevant documents, search GS or other relevant sources based on those phrases and make predictions. In addition, the excerpts selection tool needs to be updated with a new search phrase, and experts should provide a list of possible sources of data to be searched for, enabling search resources unless journals, PMC-OA or PMC are used.

## Supplementary Material

btad035_Supplementary_Data

## Data Availability

Much of the data used in this article is publically available, e.g., PubMed, GoogleScholar, and bioRxiv. But PDF of articles downloaded from Temple Universitys online system cannot be shared openly because they require paid journal subscriptions. Interested readers will need to use the scripts we have shared to download those data directly from their own subscriptions and library services.
