# Supplementary material for: Discovering research articles containing evolutionary timetrees by machine learning
Source: Bioinformatics. 2023 Jan 17;39(1):btad035. doi: 10.1093/bioinformatics/btad035 (PMC9887078; doi:10.1093/bioinformatics/btad035)
Supplement: btad035_Supplementary_Data [file btad035_supplementary_data.pdf]

Discovering research articles containing evolutionary timetrees

Supplementary materials

Marija Stanojevic[1,*], Jovan Andjelkovic[1], Adrienne Kasprowicz[3], Louise A. Huuki[3], Jennifer Chao[1], S. Blair Hedges[2,3], Sudhir Kumar[2,3,*], and Zoran Obradovic[1,*]

1 Center for Data Analytics and Biomedical Informatics, Computer and Information Science, Department, Temple University, Philadelphia, PA, 19121, USA

2 Institute for Genomics and Evolutionary Medicine, Temple University, Philadelphia, PA, 19121, USA

3 Department of Biology, Temple University, Philadelphia, PA, 19121, USA

Data Collection Process. Besides software for finding and downloading papers through Google Search, we created software that collected data from 18 journals using Temple University access. In addition, PMC datasets (PMC-OA and PMC Historical) were downloaded manually using FTP bulk download. We also developed software that uses bioRxiv API to get information about all articles published since 2013, when the server was started. Downloaded data was in PDF format from all the sources except PMC, which already contained textual files. While collecting the data, we created metadata files for each source containing the title and other available information, such as a list of authors, a link, the year of publishing, and more.

Intelligent Search. We use TFIDF and Lasso approaches to create a list of words with positive coefficients in the Lasso model**.** TFIDF has selected the following phrases (PL list) as the most important positive features: *mya, divergence time, myr, 2015, the divergence, Miocene, node, diversification, beast, million years, mybp, split, extant, diverged,* and *chronogram*. After that, we created WL using word2vec to find frequent words appearing in the text context of words from the PL list. We join those two lists into FL = PL + WL and use those words to create FL-simple and FL-complex queries.

Four FL-complex search queries are created, and they are given below. For creating FL-simple queries, we had to select phrases not often used in other sciences. In FL-complex queries, we could search for papers with more common phrases, such as "million years", if we give additional context to help the search focus on relevant papers. We have also selected different publishing years for both types of queries, focusing on research from the last two decades only.

Simple Search Query Phrases: 1) *mya, 2) Pliocene, 3) phylogenetic, 4) mybp, 5) carboniferous, 6) phylogram, 7) myr, 8) timetree, 9) mybp, and 10) treeannotator*.

Complex Search Queries (each query was run with a different span of years so that all papers from the 1990s onwards are searched):

1) species AND (ma OR mya OR myr OR divergence OR miocene OR diversification OR beast OR pliocene OR carboniferous OR outgroups OR mybp OR extant OR diverged OR treeannotator OR timetree OR kya OR million years) AND year=2016

2) (chronogram OR phylogeny OR time tree OR phylogram OR BEAST drummond common ancestor OR crown age OR dated phylogeny OR dating analysis divergence time OR fossil calibrated OR fossil calibration OR fossil record) AND year = 2019

3) molecular AND (ma OR mya OR myr OR divergence OR miocene OR diversification OR beast OR pliocene OR
carboniferous OR outgroups OR mybp OR extant OR diverged OR treeannotator OR timetree) AND year = 2018

4) (molecular clock OR MrBayes node age OR relaxed clock OR stem age OR strict clock time calibrated) AND from_year = 2009 AND to_year=2015

Journals considered. This section lists all journals from which we collected data:

1. Molecular Phylogenetics and Evolution, Elsevier
2. Molecular Biology and Evolution, Oxford Academic
3. Journal of Biogeography, Wiley
4. Evolution, Wiley
5. Systematic Biology, Oxford Academic
6. Molecular Ecology, Wiley
7. Ecology and Evolution, Wiley
8. American Journal of Botany, Wiley
9. Biological Journal of the Linnean Society, Oxford Academic
10. Zoological Journal of the Linnean Society, Oxford Academic
11. Journal of Evolutionary Biology, Wiley
12. Gene, Elsevier
13. Systematic Entomology, Wiley
14. Journal of Zoological Systematics and Evolutionary Research, Wiley
15. Annals of Botany, Oxford Academic
16. Botanical Journal of the Linnean Society, Oxford Academic
17. New Phytologist, Wiley
18. Zoologica Scripta, Wiley
19. Cladistics, Wiley

Text Extraction. PMC files were zipped together in textual format, so we automatically extracted individual files and collected the needed text. Since all other resources contained PDFs, we used the python library "textract" to get textual information. Some journals had PDFs that were scanned papers published more than a century ago, from which we extracted the text using

the python library "ocrmypdf." Conversion from pdf to text resulting in the extraction of fewer than 100 characters is excluded. Errors from downloading, such as duplicates or missing files, are also removed.

Filtering Dataset. A few filtering techniques were tested using the following criteria: 1) if the sum of occurrences count of all words in the list is greater than a threshold; 2) if more than a threshold of words from the list appears in the text; 3) if one of the words in the reduced list exists in the paper; 4) combination of criteria 1 and 2. Criterium three was chosen as it was the simplest and fastest. In addition, it ensured that only relevant papers that might contain time tree data needed for the database are chosen, and it is susceptible to bias of year published, journal, publisher, or dataset source. Reduced list required text to contain one of the strings: 1) *mrbayes*; 2) *beast*; 3) *timetree*; 4) *treeannotator*; 5) *phylogen*; 6) *beauti*; 7) *reltime*; 8) *multidivtime*; 9) *r8s*; 10) *treefinder*; 11) *path o gen*; 12) *phytime*; 13) *time tree*; 14) *phylogram*; 15) *relaxed clock*; 16) *mega*; 17) *molecular clock*; and 18) *strict clock*. Phrases that contain dashes instead of space between words are also considered.

Selecting Relevant Text Excerpts from Papers. Since texts come from different sources, including OCR transformation, they have various formatting. Sometimes, we cannot understand where the paragraph starts or ends. In those cases, we took 400 characters before and after the figure was mentioned. First, we label figures containing a timetree from 816 positively labeled papers. Then, we assign a positive label to each extracted text discussing those figures and a negative label to all extracted text excerpts discussing some other figures from those research papers. Also, we take 504 papers labeled as negative in the TT dataset and label all texts extracted from those papers as negative. This procedure results in a dataset of 7094 extracted text excerpts, 3550 positively labeled.

Experimental Settings. We normalize text by removing all non-alphabet characters and converting the remaining text to lowercase. We randomly reordered examples in the labeled dataset and split them into training, cross-validation, and testing with ratios 73:7:20, respectively, for whole papers classification and 66:14:20 for selected texts classification. For representation learning with TFIDF, we select phrases (words and word pairs) that appear in at least 5% and in at most 90% of the documents. These boundaries remove common stop words (e.g., the, a, we) and words too rare to be essential for the classification. When creating doc2vec representation, we train a neural network for 20 epochs to produce the final document vector representations of 100 dimensions. The enhanced representation learning uses the same hyperparameters.

To train BERT-based classifier models, we used the learning rate of 0.00002, as recommended by the original authors (cite). We found that training for two epochs works the best, and we used a batch size of 8 samples to overcome hardware restrictions. We use HuggingFace transformers library implementation of BERT-based models named: bert-base-uncased, distilbert-base-uncased, allenai/scibert_scivocab_uncased, and dmis-lab/biobert-base-cased-v1.1

as those are the best-performing versions of the respective models. We implement a HuggingFace transformers trainer for simple training and evaluation and a PyTorch trainer with an accelerator for more complex settings.

We use a grid search for hyperparameters to find the best classification model. Hyperparameters for text representation and classification were set based on standard practices. We multiply errors with the inverse of class size to ensure the model is equally penalized when making mistakes for each class despite differences in the number of samples available with that label. We use random state 0 for DT, RF, SVM, Ada, GB, and Bag to create reproducible results. We repeat all experiments five times, except those containing BERT, BioBERT, or SciBERT, which are repeated only two times due to limited access to the GPU hardware. We report the mean and standard deviation of the results.

Hyperparameters for classification models. Hyperparameter information not given in the main manuscript is as follows.

- **Logistic regression with L1 regularization** - regularization coefficient alpha tested on values 0.000001, 0.00001, 0.0001, 0.001, 0.01, and 0.1. Models are trained using the SGD algorithm with logistic regression loss, L1 penalty, and up to 500 iterations.
- **Decision Tree** - impurity decrease in decision node tested on values 0.000001, 0.00001, 0.0001, 0.001, 0.01, 0.1, and 1 using entropy criterion.
- **Random Forest** - impurity decrease in decision node tested on values 0.000001, 0.00001, 0.0001, 0.001, 0.01, 0.1, and 1 using entropy criterion.
- **K nearest neighbors** - k tested on values 1, 2, 3, 4, 5, 6, and 8.
- **Support vector machines** - regularization parameter tested on values 0.1, 0.3, 1, 3, 10, 30, and 100 combined with linear, polynomial, RBF and sigmoid kernels.
- **Adaboost** - number of estimators tested on values 40, 80, 160, and 320
- **Gradient boosting** - number of estimators tested on values 40, 80, 160, and 320
- **Bagging** - number of estimators tested on values 40, 80, 160, and 320

Words that are not considered when visualizing most frequent words from texts (Figures 2, 3, 4, and 6) are stopwords defined in python “wordcloud” library enhanced with**:** *'fig', 'figure', 'et', 'al', 'two', 'within', 'figs', 'three', 'table', 'found', 'among', 'using', 'shown', 'may', 'one', 'see', 'used', 'supported', 'new', 'well', 'a', 'b', 'c', 'd', 'e', 'f', 'g', 'h', 'i', 'j', 'k', 'l', 'm', 'n', 'o', 'p', 'q', 'r', 's', 't', 'u', 'v', 'w', 'x', 'y', 'z', 'dr', 'mr', 'org', 'biorxiv', 'preprint', 'peer', 'doi', 'review', 'copyright', 'version', 'http', 'https', 'author', 'license', 'international', 'certified', 'granted', 'reserved', 'posted', 'funder', 'holder', 'available', 'perpetuity*', *‘made’, ‘display’, ‘will’, ‘cases’, ‘first’, ‘hospital’, ‘case’, ‘medical’, ‘many’, ‘must’, ‘acc’, ‘allowed’, ‘without’, ‘reuse’, ‘much’, ‘upon’, ‘now’, ‘general’, ‘great’, ‘present’, ‘said’, ‘work’, ‘small’, ‘man’, ‘london’, ‘large’, ‘little’, ‘good’*

Classification results

The first column says which text representation and classification algorithms are used, the next three columns show F1 scores when representation is learned on all datasets (original TT and collected). Column two shows the results of representations trained on the text of whole papers, column three on selected figure descriptions of length around 800 characters, and column four on selected figure descriptions of length around 300 characters. We consider the best algorithm that gives the best F1 score.

Table S1. Classification results of models using enhanced representation.

	**Features from labeled and unlabeled data**		
	**Whole paper**	**Selected text**	**Small selected text**
**L1 + tfidf**	0.698 ± 0.011	0.724 ± 0.013	0.622 ± 0.042
**L1 + doc2vec**	0.637 ± 0.010	0.727 ± 0.007	0.717 ± 0.005
**DT + tfidf**	0.602 ± 0.001	0.654 ± 0.031	0.650 ± 0.001
**DT + doc2vec**	0.526 ± 0.023	0.663 ± 0.018	0.636 ± 0.019
**RF + tfidf**	0.700 ± 0.004	0.737 ± 0.011	0.650 ± 0.001
**RF + doc2vec**	0.604 ± 0.040	0.764 ± 0.007	0.727 ± 0.014
**KNN + tfidf**	0.606 ± 0.001	0.674 ± 0.020	0.584 ± 0.007
**KNN+doc2vec**	0.590 ± 0.014	0.751 ± 0.009	0.670 ± 0.001
**SVM + tfidf**	0.701 ± 0.007	0.739 ± 0.002	0.621 ± 0.003
**SVM+doc2vec**	0.672 ± 0.003	0.774 ± 0.011	0.751 ± 0.013
**Ada + tfidf**	0.700 ± 0.001	0.700 ± 0.016	0.594 ± 0.004
**Ada+doc2vec**	0.571 ± 0.001	0.727 ± 0.005	0.699 ± 0.003
**GB + tfidf**	0.700 ± 0.020	0.714 ± 0.012	0.602 ± 0.021
**GB + doc2vec**	0.574 ± 0.028	0.763 ± 0.010	0.731 ± 0.002
**Bag + tfidf**	0.681 ± 0.011	0.727 ± 0.006	0.601 ± 0.012
**Bag + doc2vec**	0.531 ± 0.009	0.750 ± 0.015	0.708 ± 0.003
